# Essay on Erectile Tumors

**Published:** 1860-06

**Authors:** Daniel Brainard

**Affiliations:** Surgeon of the U. S. Marine Hospital, Chicago, Etc.


### ARTICLE 4.

### ESSAY
### On the Pathology and Treatment of Erectile Tumors.

BY DANIEL BRAINARD, M. D., SURGEON OF THE U. S. MARINE HOSPITAL, CHICAGO, ETC.

The term, erectile tumor, is employed to designate that form of disease known under various names of *aneurism by anastomosis*, *nævus maternus*, or marks, *telangeictasis*, etc.—The term, although liable to objections, is not more so than any other, hence its general adoption.

This disease, notwithstanding its frequency, seems to have been confounded by surgeons with fungus and cancerous diseases, until the latter part of the eighteenth century, when it was noticed by J. L. Petit,* under the name of varix ; and by John Bell,† to whom we are indebted for the name of aneurism by anastomosis.

Tumors of this kind have been divided according to their situation, into cutaneuos and subcutaneous ; according to their characters, into arterial, (*aneurism by anastomosis*) venous, (mostly subcutaneous,) and capillary, nævus, (marks) and such as are mixed, partaking of the arterial and venous characters, or occupying a superficial and deep situation at the same time. Some of the French writers have given the name of *fungus hæmatodes* to this tumor, and seem to have

---

*Traite des Maladies Chirurgicales, Vol. 1, p. 232.
†Principles of Surgery. p. 102.

attached the idea of malignancy or liability to degeneration to it. No well authenticated instance of such degeneration has, perhaps been adduced, and the term, fungus, in never applied to it at the present day. There are, however, points where the two diseases, erectile and cancerous, seem to touch each other.

A child, eighteen months old, was brought to me from Wisconsin, in 1849, with a mark of a dark blue color extending over the upper part of the right thigh, hip, side of the trunk to the axilla, and before and behind to the median line. Upon the side of the abdomen rose a tumor of the size of a man's fist. I removed it and found it composed of perfectly formed cerebriform matter throughout. The disease re-appeared in a few weeks, and the child died of it soon after.

The color florid, blue, and of various intermediate and intermingled shades, the great diminution or entire disappearance under pressure, the pulsations and vibratory thrill in the arterial variety, are of themselves sufficient to diagnosticate this disease There are cases,however, such as those reported by Mr. Liston* and Mr. Birkett,† where a fibrous element or the presence of adipose tissue may render a diagnosis difficult or impossible, without resort to the exploring trocar. These tumors, when superficial or venous, are most frequently congenital, often numerous on the same subject, and given to occurring in certain familes.

The pulsating tumor originates at any age, and most frequently from violence.

The structure of these growths has been the subject of very careful investigations. Nelaton, A. Berard, Liston, Birkett, Wardrop, Paget, Rokitansky, Haike, and many others, have published accounts of their dissections. I have myself, examined a large number after extirpation. They have, in some cases, a striking resemblance to tissue of the corpus spongiosum urethræ. A probe penetrates the tissue, and

---

*Med. Chir. Trans. vol. 26, p. 125.

†Ibid, vol. 30, p. 193.

water injected passes through it in every direction. Mr. Paget describes the subcutaneous variety perfectly, as follows: "The common structure of this form of tumor, is a collection of minute blood vessels, dilated and closely arranged within a limited area of some natural texture."* There are, however, peculiarities in the structure of all the growths, which separate them from varix, and from simple enlargement of the capillary vessels. This is demonstrated by the tendency which they have in some cases to disappear, in others to remain stationary, in others still, to increase with rapidity. If, on the one hand, the microscopic characters of a tissue are useful in determining its "pathological physiology," on the other, its history is far more important in determining its intimate structure. Guided by the well known nature and tendency of such growth, we are not only authorized to retain them as a distinct class, but to establish them among subdivisions and distinctions broader and more marked than those commonly recognized.

The latest and best pathological researches seem to accord entirely with this view. Rokitansky, in his treatise on pathological anatomy, and more recently, in an essay presented to the Academy of Sciences of Vienna, has described these growths under the name of "cavernous structure," and as "products of new formation." "The stroma of erectile tumors," he says, "is formed by a network of fibres analogous to those of cellular tissue, the thickness of the septa being very variable. From the thicker septa there radiate others more delicate which incompletely circumscribe irregular spaces which communicate with each other."†

Birkett, in his description, says they must be classed with the fibrous tissues nourished by arteries and possessing cells which are in communication with the larger or smaller veins.‡ This account agrees entirely with that given by Haike, of the structure of the tumor of the breast treated by Mr. Image.

---

*Surgical Pathology. p. 481.

†Am. Journal of Med. Sciences for 1855. p. 231.

‡Loc. Cit.

Of the superficial variety, there are a great number whose tendency is to disappear soon after birth. Nothing is more common than to meet in new born infants, marks about the face and neck especially, which not only disappear, but which, to the careful observer, bear marks of being at birth in the course of diminution. They yield to the mildest treatment.

The subcutaneous venous tumor has little tendency to change; it grows or diminishes in size but slowly, and is cured with difficulty. Its analogy to varix, hemorrhoids and varicocele, in all of which the obliteration of a vein is extremely difficult, is apparent.

The arterial variety has a tendency to increase rapidly, pulsates, has the creeping thrill, called by the French, *susurus*, and seems to attract blood to all the neighboring parts, rendering the veins and arteries turgid, injecting the capillaries. The case of Mr. Wells, hereinafter reported, and a case related by Dr. Warren,* are illustrations of this tendency. The analogy offered by such tumors to aneurismal varix cannot be overlooked. They are frequently not congenital, occur as the effect of wounds, and are more subject to treatment than the venous tumors.

*Treatment.*—If, as has been asserted, the number and variety of remedies proposed indicate the difficulty of cure, there is no disease more obstinate than the one under consideration. The following may be considered as the principal, arranged in the order of their usefulness: 1, excision; 2, strangulation; 3, compression; 4, cauterization; 5, setons or needles and injections; 6, ligation of the principal artery leading to tumor. To these may be added several others, of doubtful utility, as the application of astringents, incisions, subcutaneous laceration, vaccination, irritating injections, galvano puncture, etc.

If the multiplicity of means is somewhat puzzling when thus enumerated, this is not the case when they are considered in individual cases.

---

*Warren on Tumors.

EXTIRPATION.

Extirpation is to be preferred whenever the tumor of sufficient size to require an operation, is so situated as to allow of this being completely or in a great measure effected without leaving behind too great deformity, and when it can be done with safety.

The apprehension of danger from hemorrhage in such operations, is, for the most part, not well founded. It is true that fatal cases have occurred, but they are rare, and no operation is entirely exempt from danger. Mr. Wardrop, in whose hands a child died from hemorrhage after excision, (a case so universally quoted against the operation) tried, in another very similar case the ligature of the carotid, without a more favorable result. The patient died on the thirteenth day.*

The following cases will illustrate my own practice:

Case 1. A child, eighteen months old, was brought to me in April, 1850, with an erectile tumor on the top of the left shoulder, half the size of a hen's egg. It had existed from birth, was of a bright scarlet color, except in some points where there were appearances of cicatrices, was slightly pulsating.

I removed it, cutting in the sound skin, performing the operation rapidly, and fetching the edges of the wound together with stitches. Little blood was lost.

Case 2. A child, three years old, was brought to me Dec., 1856, with an erectile tumor of the size of a hen's egg, situated on the anterior margin of the left axilla. It was of the same character as the preceeding, congenital, and had been transfixed with ligatures, and, I think, needles, without benefit. The erectile tissue in this case passed deeply into the axilla, the pulsation was very perceptible, and the surgeons who had been consulted, were deterred from attempting extirpation.

I transfixed the tumor at its base with fine needles, leaving the ligatures in. I then excised it and had the ligatures tied

*Med. Chir. Transactions. vol. 9, p. 206.

as quickly as possible although all the tissue was not removed. The amount of blood lost was by no means great. The wound healed by granulation, and the cure is perfect.

This case is an illustration of the advantage of removing a part where all the tissue cannot be reached. The following is also in point.

Case 3. A Jewish child was brought to me in the Spring of 1853, with an erectile tumor situated on the top of the forehead and extending into the hairy scalp. As the removal of the whole would have left an unseemly scar, I transfixed it at the base with needles, leaving ligatures behind, removed a part of the mass embraced by the ligatures and tied them firmly over the rest. This tumor was mostly subcutaneous, the ligatures were left in place until suppuration occurred and the cure was perfect.

I have removed these tumors by excision from almost every part of the head, trunk and members, without accident.—There are, however, many situations, in which this operation would be dangerous and in which they cannot safely be removed. Of this kind are erectile tumors of the tongue, those extending into the nasal or buccal passages, deep seated, large sized tumors, situated about the neck, etc.

STRANGULATION.

In many of these instances, ligation, so as to strangulate, is the best treatment.

Case 4. A new born infant was brought to me, with an erectile tumor as large as a small strawberry on the end of its tongue. I transfixed the tongue at the base of the tissue with a needle armed with a double ligature, and tied one thread on each side in such a manner as to embrace the whole. Sloughing occured and a perfect cure was effected.

Case 5. A child 12 years of age was brought to me, with an erectile tumor on the right side of the face, embracing the upper lip from the median line, the cheek, angle of the mouth and a part of the lower lip. The disease was quite too extensive for extirpation. I attacked it with needles, inserting first three horizontally through the upper lip and placing ligatures firmly upon them. The needles were so placed as to embrace nearly the whole substance of the lip—at the end of six days they had nearly cut through and were removed. After cicatrization had commenced I inserted four needles vertically in the cheek, and placed ligatures on them in the same manner; these were removed the fifth day. A small portion of tissue which remained below the angle of the mouth was embraced in two ligatures, firmly tied. At the end of five weeks from the first operation scarcely a trace of the disease remained. The cicatrix was not unsightly.

Case 6. A child six years old was brought, with a tumor of the venous kind situated at the external angle of the eye, and extending into the orbit and along the upper lid to its centre. It was as large as a hickory nut. Fearing the deformity which removal might leave, I transfixed it with four ligatures as near the base as possible and tied them moderately tight. Considerable inflammation followed and an abscess formed in the tumor. At the end of a week the threads were removed, the inflammation subsided, and the tumor was effaced.

The means of effecting strangulation are as various as the cases we are called upon to treat. Ligatures, single, double, or more numerous, passed through the base of the tumor, and tied—the same passed at a right angle or under the skin, so as to effect subcutaneous strangulation, have recently been tried. Needles may also be used singly, in pairs, or in greater numbers, placed parallel or at a right angle, with each other. Upon these, ligatures may be applied in the form of a figure eight or around the base of the tumor, modified according to the necessity of each case.

Recently, Chassaignac's *Ecraseur Liniere* has been used for strangulating these growths. Mr. Mayor, in his work entitled "ligature en masse," recommends an instrument like a tourniquet. Græfe, as long ago as 1833, invented an instrument for strangulating tumors of all sorts, and the use of ligatures with canulæ in certain cases is a practice well known to the profession.

The first case I find treated by ligature, is in La Motte

*Traite conplet de chirurgie*, vol. 1, p. 333, 1761. Recently, every one has used the ligature in some form.

I have used also strangulation with advantage in connection with other means, cases of which will be given hereafter.

COMPRESSION.

The cases of erectile tumor, cured by compression, are but few, and it is a means which fails far more often than it succeeds. The cases to which it is adapted are those of small size, situated on the surface of the skull. Such tumors, and many others situated on different portions of the cutaneous surface, have, in some instances, a tendency to disappear spontaneously. Hence it is better not to be in haste about an operation in any case, but take time to observe its tendency in this respect. This counsel, approved by most judicious surgeons, has been especially insisted upon by the very distinguished obstetrician, Moreau, of Paris, and should not be forgotten.

In the mean time we can resort to compression and may gain the credit of effecting a cure which would have occurred spontaneously. The best method of using compression, is, in my view, that which I introduced into practice in 1849, viz., the application of collodion, which, by contraction on drying, effects the object and expels the blood in the simplest manner possible.

Case 7. A child about a year old, had an erectile tumor of a bright red color, but not pulsating, of the size of a large strawberry, situated over the anterior fontanelle. I advised the application of collodion, which was repeated as often as it became loose. Three applications completely removed it. This case is noticed in the report of Prof. R. D. Mussey, made in 1851, and published in the Transactions of the Am. Med. Ass., for that year.

Case 8. A new born infant was brought to me in 1851, with a superficial tumor, situated under the left eye, about one-fourth of ah inch in breadth and extending the length of the lower lid. The collodion, being advised, was applied during sleep. Owing to the extreme mobility of the parts, the pellicle formed was easily detached and the pressure less effectual than in cases where the tumor is situated over bone.—However, at the end of a year, scarcely a trace of the tumor or discoloration remained.

These are cases taken at random from my note book, as illustrating the forms of the disease in which I have found this simple method of treatment effectual. I might add many others in which it was of no avail; they were, in general, those of larger size and deeply situated.

Compression has been recommended by Pelletan, Boyer, Abernethy, Dupuytren, and others, yet it has not at the present day the confidence of any surgeon.

Boyer's case, where a mother, by repeatedly pressing on the tumor, situated on the lip of her child, cured it, is unfortunately alone in science, and is to be ranked, perhaps, with those of spontaneous disappearance. It is generally agreed that if compression is to be tried at all it should be in those cases only where the tumor is superficial and situated over a bony surface, and that it should be applied by means of a spring or firm bandage. But even under the most favorable circumstances I should not recommend a resort to it except as related above.

### CAUTERIZATION.

This may be affected by the actual cautery, by the potential cautery, and by the various milder forms of caustic.—The actual cautery is probably the most ancient method of destroying vascular growths; indeed it was almost the only means known to the older surgeons. As they seem not to have been distinguished from other tumors, its application to them is not positive, but from the notice of its uses in fungus of the antrum and tumors about the anus, there seems but little doubt thas it was used in vascular tumors.*

Without entering further into the subject it may be sufficient simply to state that as a means of *destroying* the morbid growth, it is, and ought to be, entirely rejected from surgical

*Consult Percy Pyrotechnie Chirurgicale Practique.

practice. As a means of effecting the transformation of vascular tissue by inflammation, according to the suggestion of Lallemand, it still retains a permanent place. Among those who have recently recommended it, we may may mention Dr. Mott, and Dr. Knight, the distinguished professor of surgery in Yale College. Guersent, surgeon of the Children's Hospital, Paris, states that he has used it successfully in about twenty cases. It is greatly to be regretted that the peculiar cases to which it is adapted, and the method of using it, have not been given, as they are points still unsettled, and no one is more capable of throwing light upon the subject than M. Guersent. Velpeau speaks highly of it and calls it the "American method."

The instances in which I have used the cauterizing needles with advantage are such as pulsating erectile tumor of the orbit, (a case of which is given further on,) and those involving the thickness of the lip or of the tongue.

It has appeared to me that whenever the morbid tissue is to be transformed by the effect of inflammation, it should be excited first upon the sound parts. This, no doubt, was the view of Dr. Stevens, who, in using setons, placed them in the sound tissue, beneath the tumor.* I have found that when the hot needles were passed through a certain thickness of sound before entering the diseased tissue, when the needles used were large, and when several punctures were made near together, the effect was decided and beneficial.— On the other hand, the erectile tissue is with difficulty thrown into such a state of inflammation as to modify it. This has been particularly insisted on by Watson, who ascribes it to the slight degree of nervous action going on in such growths and I believe that both their sensibility and nutrition are of low grade. Certain it is, that inserting hot needles in them has little effect except the sound tissue be also touched.

Mackilwain seems to have been the first to resort to this form of cauterization. As an auxiliary, it is a valuable

---

*Watson on Telangiectasis. American Journal of the Medical Sciences, method of treatment; alone, I think there are few cases where it will be found sufficient.

Caustics, of various kinds, such as the potassa fusa, the solidified sulphuric acid, the acid nitrate of mercury, have been resorted to for the destruction of vascular growths.— They have, however, no advantage over the knife in most cases. When the surface to be affected is extensive, a feeble caustic is very useful. The application of strong nitric acid is practised with advantage in many cases of nævi. It forms a solid eschar which does not separate for many days. The other means for exciting inflammation are such as inserting needles, pins, setons, injections of various kinds, etc.

Of these, the insertion of metallic needles of any kind, is the least reliable, and ought, perhaps, to be entirely rejected from practice. This arises from the well known fact that metallic bodies may sojourn indefinitely in the living tissues without causing much inconvenience. Berard,* in his well known and valuable essay on this subject, states that he had often seen needles come out quite dry after having remained several weeks in such tumors. Those who may be disposed still to think favorably of acupuncture or other needles, I would refer to the essay of Berard, whose observations seem to me conclusive against them.

Setons are scarcely more reliable than needles. If a thread is simply passed through, it produces little effect and the solidification is not permanent.

If the lighter forms of caustic are introduced upon the thread, as nitrate of silver, the action is more prompt but not more effectual. The use of caustic potash or mineral acids on the threads is dangerous, as these substances pass directly into the circulation. The same objection applies to the injection of caustics into the openings left by the threads of the seton, as has been sufficiently shown by Berard.

These objections do not apply to either the pins or ligatures, when they are so used as to effect very perfect stran-

---

*Gazette Medicale for 1841.

gulation of the tumor. In illustration of this part of the subject, the following case will suffice:

Case 9. Miss A., aged 18 years, applied to me, Aug., 1856, with an erectile tumor, of the venous cubcutanous kind situated under the left eye.

I commenced the treatment by transfixing it at its base horizontally, with four needles, upon which ligatures were placed with moderate tightness. At the end of seven days these were nearly cut through and the tissue between the skin and needle divided; they were therefore withdrawn.— The tumor became inflamed, with several little abscesses in its substance. There was only a small part upon the surface of the lower eye lid which was still elastic. This was transfixed horizontally with a needle armed with a ligature which was tied moderately tight. This, at the end of eight days, had nearly divided the tissues which it embraced, and was removed. Collodion was then directed to be applied over the whole surface, and renewed as often as it separated, and the patient returned home. The tumor, at that time, seemed very firm and I entertained little doubt that a perfect cure would be effected.

At the end of three months the patient returned, with the tumor very nearly in the same state as before the operation. The marks of the former operation were readily perceptible. I then advised excision of a part of the tumor, but she objected to the use of the knife, and I transfixed it at the base with three setons, placed transverely. They were of silk, and previously dipped in a solution of nitrate of silver.—one drachm to the ounce of distilled water. These were not tied but left in, and allowed to remain four weeks, at the end of which time they were nearly ulcerated out, and were removed. They had, however, produced much less inflammation than the needles alone, and the elasticity of the tissue was scarcely diminished. The patient still objecting to excision, I covered, March 5th, 1857, the whole surface with the caustic composed of nitric acid solidified with saffron. This was allowed to remain about five minutes, until it had cauterized the skin superficially. The cauterized surface was dressed with simple cerate. This cauterization was too superficial to produce any decided effect, and accordingly, March 11, 1857, I applied the caustic potash to the surface one-half an inch in breadth and over an inch in length, until an eschar was formed. As soon as the surface was penetrated, hemorrhage occurred and the application was stopped.

April 29th.—Eschar separated and cicatrization nearly complete. Tumor much diminished in size, but the elastic tissue persisting. Re-applied the potash upon the same spot so as to cauterize deeply. An eschar formed and was detached—cicatrization was completed, but the tumor was still scarcely affected.

July 5th, 1858—I excised it in part, taking care to preserve all the skin. The tissue seemed entirely like that of the corpus cavernosum.

The combination of the seton and caustic has been proposed. Prof. N. P. Smith, of Baltimore, relates (*Maryland Med. and Sur. Journal*, March, 1853,) a case in which he passed threads, dipped in a solution of caustic potash and dried, through the base of an erectile tumor. The case is not given in detail, and seems to have been still under treatment when the report was made.

For myself, the fear of the toxic effect of introducing caustic potash into the circulation, has made me hesitate before imitating this practice.

M. A. Berard, in the essay already referred to, relates several cases in which, after using the seton, he injected the acid nitrate of mercury, the effects of which, in some cases, were favorable and in others gangrene, and constitutional symptoms of poisoning resulted.

### INJECTIONS.

This method of treating erectile tumors is attributed by Watson, to Samuel Cooper, and is said to have been successfully employed by Mr. Lloyd. The injection consisted of from three to six drops of nitric acid dissolved in a drachm of water—it was thrown into the tumor by means of a syringe through a minute puncture near its base. During the operation careful pressure is made in all directions around the tumor to prevent the fluid from entering the general circulation.

The next case on record, in which this practice was employed, was attended with an instantaneously fatal result.* These cases were published in 1836–37. Bell is said to have used it also with success, and Messrs. Toogood and Ward unsuccessfully.

Velpeau, in the second volume of the second edition of his Operative Surgery, says, "I should not hesitate to inject sub-cutaneous tumors, employing a larger syringe and tincture of iodine in place of the solution of nitric acid. He did not put the suggestion in practice, and seems to have intended the tincture to be drawn off as in the operation for hydrocele. I put this sguggestion in practice.

Case 10. There were several venous tumors situated on the anterior part of the right thigh. They were elastic, capable of being removed by pressure, and composed of tortuous and convoluted veins. The patient was a man of fifty-four years of age, and his constitution was broken by repeated attacks of disease and a fracture of the right femur, which had never united. Assisted by Prof. Herrick, I threw into one of these tumors two grains of iodine and six grains of iodide of potassium in two drachms of water. The injection was made with Anel's syringe through a small exploring trocar introduced to the center of the tumor.

Almost instantly the patient began to spit and continued discharging saliva freely during three days, complaining all the time of a bitter taste in his mouth. The tumor, after the operation, became more tense, and warmer than natural, and after the fifth day contracted below its former size and was flaccid.

Encouraged by these results, I repeated the injection a second time, one week after the first, with the same results in every respect. This case is noticed by me in the Northwestern *Med. and Sur. Journal*, 1850.

---

*Watson, Amer. Jour. Med. Sciences. vol. 24, p. 48.

The declining state of the patient's health was the only cause why the treatment was not persevered in. The solution in this case passed directly into the circulation, producing instantaneous iodine ptyalysm, and was not retained in the tumor as it should have been.

Such was the state of our knowledge on this subject in 1853. There was little to encourage repetitions of the trials of the solutions hitherto tried. Tincture of iodine must, in such cases, be more dangerous than the solution, from the alcohol which it contains, and no one would dare to throw it into the current of the circulation.

It was in January, 1853, that the suggestion of Pravaz, of treating aneurisms by injections of the perchloride of iron, was laid before the Imperial Academy of Medicine, of Paris. This plan, in so far as it related to aneurisms, does not concern us here. It has, however, been applied to erectile tumors, and if it is to be preserved as one of the established operations of surgery, it seems likely to be in its application to these rather than aneurisms. Indeed, the first case treated in thls way was obviously an erectile tumor and not an aneurism. The case was reported to the Society of Surgery, of Paris, on the 30th of March, 1853. The operator was Mr. Raoul, and the tumor a supposed aneurism of the infraorbital artery. (See *Gazette des Hopiteaux* for 1853, page 177.)—Ten or twelve drops were at different times injected, and the effect seemed at first favorable, but about the time a cure seemed to have been effected a new tumor of the same description made its appearance.

October 3d, 1854, Mr. Follin read to the Society of Surgery the report of a case of erectile tumor of the face, affecting the gums, lips, cheek, etc. Injections of the perchloride of iron were made into the lip at two different points. The immediate effect was the production of hard knots at each point. In five days the lip was very much swollen; one of these points suppurated, and at the end of a month no trace of either remained. Several injections into the substance of the gums and the veil of the palate where the parts were diseased, resulted in ulceration in three days. (*Gazette des Hopiteaux* for 1854, p. 491.

January 18th, 1854, Mr. Giraldes presented a patient to the Society of Surgery, on whom he had injected a venous tumor on the side of the neck, of the size of a nut. Whether this tumor was erectile or a simple varix, does not appear, but I judge the former, as a simple enlargement of the veins of the neck would scarcely require such active treatment.—An injection of perchloride of iron was made which had the effect, at first, of greatly increasing the size of the tumor, which, however, sixteen days after the operation, was diminished in size. (*Gazette Medicale* for 1854, p. 54.)

The *Medical Times amd Gazette*, for August, 1857, says, " during the last two months we have witnessed several trials of the acid solution of the perchloride of iron as an injection for producing the coagulation of the blood in nævus, etc. In more than one instance very severe inflammation of the part has followed its use, and in one, a nævus of the scalp, sloughing and even exfoliation of a portion of bone resulted."—(*Am. Jour. of the Med. Sciences*, for Oct., 1853, p. 510.)

Mr. Foster reported two cases of erectile tumor, treated by injections of the chloride of iron. Both are reported cured.*

Mr. Riberi injected aromatic wine into a tumor of the venous subcutaneous kind, situated on the neck. Careful compression was made round it to prevent the wine from entering into the circulation. A cure was effected without gangrene.—*Am. Jour.*, vol. 38, p. 207.

Tyrell was in the habit of injecting erectile tumors with a solution of alum, using Anel's syringe. After two or three injections they hardened and shrunk away.—South in *Chelius Surgery*, vol. 2, p. 565.

The idea of injecting so energetic a caustic as the concentrated perchloride of iron into the centre of an aneurism or an erectile tumor, is certainly not in accordance with all our preconceived notions of judicious surgical practice. The

*Braithwaite's Retrospect, part 29, p. 206

blood, even if coagulated by it, could only be regarded as a foreign substance.

Experience speedily confirmed what sound theory would have led us to expect, and the practice has fallen into disuse both in erectile tumors and aneurisms.

At the time Pravaz was experimenting with the perchloride of iron, I was pursuing a similar course of investigations concerning the solution of the lactate of the same metal.—On the 14th of December, 1852, twenty days before the first communication of Pravaz's experiments with the perchloride of iron on animals, and long before Mr. Raoul had first tried it on the human subject, I had used the solution of the lactate successfully on a case of "aneurism by anastomosis" of the orbit. I had not thought of effecting the coagulation of the blood in the vessels, but desired to produce adhesive inflammation of the erectile tissue and effusion of inflammatory lymph. Having learned, from the experience of others, that acids were dangerous if passed into the blood; and from my own, that iodine was ineffectual, and perhaps not free from danger, I experimented with a large number of solutions with a view of finding one at once capable of exciting inflammation and innocuous when thrown into the circulation. The solution of the lactate of iron seemed more nearly to possess these properties than any other made use of, and accordingly it was selected in the case referred to.

As this case presents an interest in other particulars, the report of it, as originally published in the *London Lancet*, is inserted here. I need only add that the cure has remained permanent, and that at the present time, June, 1860, Mr. Wells is a healthy man, attending daily to a large business.

*Case of Erectile tumor of the orbit, cured by infiltration with the solution of the Lactate of Iron and puncture with hot needles, after the ligature of the carotid artery had failed; with observations on the effect of that solution in obliterating the bloodvessels.*

William W——, a farmer, aged thirty-four years, of good constitution, consulted me, August 1st, 1851, on account of a tumor of the left orbit. The eye was prominent, but could be closed; it rose perceptibly with the pulsation of the arteries, and gave a thrill on being touched. On placing the ear in contact with the tumor, a loud blowing sound was heard, which could be perceived, but less distincty over the whole head. The veins of the face were prominent; the head was hot, and the arteries of the neck and head pulsated with unnatural force. Severe pain was often felt in the head, attended with nausea and vomiting; pressure on the left carotid instantly stopped the pulsation and sound.

*History.*—On the 14th of July, 1851, the patient received a severe kick from a horse on the left side of the lower jaw, which fractured it on the right side, and otherwise severely injured him. On recovering from the shock, he first perceived the sound in his head. This circumstance was calculated to mislead the surgeon in the opinion of a traumatic aneurism; but on a careful inquiry I found that the left eye had long differed from the other, principally in being short-sighted. Finding compression could not be borne on account of the great tenderness, I advised the ligature of the left common carotid artery; but the patient's mind being unprepared for so serious an operation, he returned home, with the direction to adhere to a rigid diet, and the application of ice or iced-water. Instead of strictly following this advice, he consulted various practitioners, regular and irregular, using by their direction every kind of application. About the 1st of November following, he returned to me. The disease was greatly aggravated. The globe of the eye was protruded, the lids could not be closed, the conjunctiva was ulcerated, and the thrill could be clearly heard over the whole of the head. His general health had suffered much, and he has had several attacks, which he called billious, and for which he had been purged and kept upon a strict diet for many days. Vomiting had increased the size of the tumor; he had severe pain in his head, and could only get relief from large doses of morphine. From the great heat in the head, he only slept by having cloths dipped in cold water kept constantly on it. He was now anxious to have the operation performed, which was done on the 11th of November, in the usual manner, the artery being tied about an inch below the division. No unusual symptoms occurred. Immediately after the operation the pulsation and sound in the tumor ceased.

The next day, the 12, he complained of some pain in the right side of the head, and the capillaries of that side became greatly injected from the effort to establish the collateral circulation. A blood-letting of eight ounces, gave great relief. The ligature came away on the fourteenth day. Immediately after the operation compression was attempted upon the tumor; but so acute was the sensibility that nothing more than the weight of a fold of linen could be borne. Bags of pounded ice and salt were applied around it, and evaporating lotions upon it. Under this treatment, which, with a rigid diet, was continued, the tumor diminihed in size; still on the third day after tying the artery, a careful examination detected a slight pulsation and sound in the tumor. This continued, the tumor still subsiding. He left me on the 10th of December. His health continued poor, and he was not able to attend to his business for several months. He noticed the sound of the circulation occasionally after the operation, and it gradually became more constant and stronger. He was often seized with what he called a bilious attack, which continued a day and a half, and was attended with severe retching and vomiting; at such times there was a marked increase of the pain and swelling, which thereafter became constant. In October, 1852, the cornea had disappeared, the conjunctiva projected in a fungous shape, and several hemorrhages occurred, from rupture probably of the superficial vessels.

On the 11th of November, 1852, just one year from the first operation, he returned to me. At this time the entire orbit was filled, the lower lid was concealed by the fungous projecting, the eye pressed outwards and downwards, and at the root of the nose and inner part of the superciliary ridge there was an elastic swelling, which had caused the absorption of the bone. It was at that point that the pulsations were strongest, and the trill most distinct. The small vessels of the forehead and side of the nose were greatly enlarged, pulsated strongly, and the latter gave the peculiar thrill of the disease very sensibly. His general health was much impaired, and he had been able to come to the city by railroad only with great difficulty.

The question of the proper treatment to be employed in a case of erectile tumor of the orbit, when the ligature of one carotid has failed to effect a cure, is one of some difficuly.—The attention is naturally turned to the other carotid; but even if the ligature of that were likely to succeed, the risk in this case was too great, since it was found that the compression of it for a few seconds rendered him perfectly insensible. None of the means resorted to for the purpose of obliterating this vascular tissue had ever been tried on deeply seated tumors like those of the orbit. On carefully reviewing them all, it was determined to try puncture with hot needles. This was done first on Nov. 13th, 1852; the needle used was the size of a common knitting-needle, sharpened to a triangular point, and set in a handle of bone. After being heated in the flame of an alcohol lamp, it was plunged into the tumor about an inch from the root of the nose, in the course of the superciliary ridge, and carried downwards and backwards to a depth of over three inches; on withdrawing it, some bleeding followed, which was readily stopped by slight pressure.

For two days there was little pain or swelling; but on the third day the inflammation was acute, the swelling extending over the face, and presenting an erysipelatous appearance.—On the fifth day this began to decline, and in a week was nearly gone. While the inflammation was at its height the tumor was more firm and the thrill less distinct, but as it subsided the elasticity and pulsation rapidly returned.

Nov. 25th.—I. repeated the opeartion, making the puncture half an inch nearer the nose, and carrying it to a depth of only one inch. The effects corresponded with those before descibed, but were a little less violent. On the third day the sound was diminished, and the firmness of the tumor very sensibly increased. On a careful examination it was found that the morbid tissue extended over the bridge of the nose and down to the inner canthus of the right eye, where a thrill could be distinctly felt.

Dec. 2d.—A puncture was made on the left side of the nose, the needle being carried obliquely upward and to the left side. The inflammation resulting from this puncture was acute, and a suppuration occurred about it. The thrill upon the right side of the nose quite disappeared.

The effect of these three punctures was to limit the spread of the tissue upon the forehead and nose; but the inflammation, although acute and extensive, was superficial, and the mass of the disease at the center was still unaffected. It was evident that the needles cooled in passing through the tissues, so as not to cauterize much below the surface.

It was therefore determined to change the treatment, and inject a fluid into the centre of the tumor capable of effecting its obliteration. For this purpose a solution of the lactate of iron, of the strength of eight grains to one fluid ounce of distilled water, filtered through paper, was preferred. The reasons for believing in the safety and efficiency of this remedy will be given below.

14th.—I punctured the tumor at its most prominent part with the infiltrating canula, carrying it to the depth of about an inch; on withdrawing the stylet arterial blood followed. A fluid drachm of the above-named solution was immediately thrown in with a small syringe, constructed for the purpose, and the canula withdrawn. The immediate effect was an intense pain in the left temporal region, and a flushing of the face, which latter only lasted a few seconds. A chill followed, accompanied with nausea and vomiting. Reaction took place in an hour, but the vomiting continued, and for twenty-four hours all drinks were ejected; pulse 63.

15th.—Vomiting continues; pain less; upper lid much swollen; pulse 65.

16th.—Vomiting less; no pain; has slept well; pulse 60; swelling increased, and so tender as not to bear the slightest touch.

23d.—For the last six days the vomiting has gradually diminished; the pulse is natural; the tumor is less tender, firm, and the pulsation perceived only at the external angle; frequent lancinating pain is felt in the orbit.

During the whole of this treatment, both of the punctures and infiltration, the head has been kept enveloped in bladders filled with a freezing mixture of ice and salt, which was very grateful to the patient. He now began to complain of its being too cold. The heat in the head was reduced to the natural standard, and from the time of the infiltration neither thrill nor sound has been perceived. The veins of the face were much diminished in size, and the pulsation of the arteries reduced to its natural state. A slight pulsation was still perceptible at the external angle of the eye, for which a puncture was made at that point with a hot needle, on January 4th, 1853.

Jan. 10.—From the time of the last puncture no pulsation has been perceived, the swelling subsiding. At this time an opening was found to exist on the anterior surface of the globe of the eye, which still remains protruded between the lids. This was followed by severe inflammation of the globe, which lasted several days. The discharge from the opening was at first the humors of the eye, afterwards pus, but no blood.

Feb. 5th.—Swelling gradually subsiding; tumor firm, no pulsation; but little pain. The patient slept, for the first time in more than a year, without some one to keep wet cloths upon the eye, dressed himself, and walked about the house; health good.

March 5th.—Swelling entirely disappeared; the globe of the eye perfectly collapsed; lids closed.

June 6th.—The patient has been pursuing his ordinary occupation for three months. His health appears perfectly restored. The left orbit seems entirely excavated and free from disease.

At this time he was presented at the annual meeting of the Illinois State Medical Society, and examined by a large number of its members, who concurred in the opinion that the cure might be regarded as perfect.

*Remarks.*—It will be readily understood that so serious an operation as throwing a fluid drachm of a solution of the lactate of iron, at a point almost to saturation, directly into the current of the circulation, would not be ventured upon without the most satisfactory reasons for regarding it, first, as safe; second, as effectual. I propose now to offer, briefly, some of the reasons for so regarding it.

In 1850 I was engaged in making inquiries concerning the effects of this salt in certain diseased states of the system, and was induced, from certain theoretical views, to try the effect when thrown into the veins. Before doing this upon the human subject, I tried it upon a dog, dissolving, rather imperfectly, ten grains of it in one fluid ounce of distilled water. This was thrown at once into the saphena vein of a middle-sized dog, and the experiment was twice repeated without any ill effect.

In December, 1851, I first used it upon the human subject, using, in the course of eight weeks, nineteen grains of the salt in solution. The largest amount injected at one time was three grains, dissolved in three drachms of distilled water. The number of times it was employed in this subject was nine. It had no ill effects, but the contrary. What is pertinent to our present subject is, that each of the veins into which it was thrown at the bend of the arm, was found, after a certain length of time, to be obliterated and converted into a firm cord, without there being produced either pain or perceptible inflammation. I have since used it in four other cases—in two of them once, and in the other two twice each—without any unfavorable effects. In one of these cases, in which it was used twice, a vein was opened a second time, three days after the first operation, but it was found filled at that point with coagulum, which, of course, was not disturbed, but the injection made by another vein. Its tendency to obliterate the veins by a gradual process of thickening their coats and obstructing the circulation, was thus made sufficiently apparent, while its safety, used with requisite precautions, was clearly demonstrated. As these cases related solely to the veins, before using it in the case of tumor of the orbit, in which it might enter directly in the arterial circulation, it was thought proper to try it upon an artery in a dog. Accordingly three grains of the lactate dissolved in three fluid drachms of distilled water, were thrown into the carotid artery of a small dog, and the vessel tied. The only effect was a slight irregularity of the action of the heart, which continued but a few seconds. As this entered directly into the cerebral circulation, its safety, when used in a much smaller quantity in the human subject, might legitimately be inferred. The result, as used upon the tumor, fully justified its employment, although the effects produced were somewhat severe. They were greatly less so, and less dangerous, than those resulting from the ligature of the carotid artery; and used in smaller quantity, repeated if necessary, it seems to promise relief without the necessity of resorting to that operation.

I may add here, that I have since used the same solution upon an erectile tumor of a venous character, of the size of a pigeon's egg, upon the inside of the lower lip, by injecting it at three different times, without any other effect than a slight tenderness, followed by gradual diminution of size and induration of tissue; but the cure is not yet complete.

The applicability of this practice to the treatment of varicose veins and aneurisms will readily suggest itself, but further experiments will be required to determine to what extent it can be relied upon in these cases. The number of the *Revue Medico-Chirurgicale de Paris* for May, 1853, contains some interesting experiments by M. Pravaz, M. Giraldes, and M. Debout, on the coagulation of the blood in the arteries of animals by the injection of a solution of the perchloride of iron, and also three cases in which it had been used for the treatment of vascular tumor in the human subject, in each of which suppuration, and in one, gangrene, followed. These results are not of a nature to encourage further trials of that substance; but if we take into consideration that the solution of the perchloride of iron is a substance foreign to the normal constitution of the blood, and produces instant coagulation when brought in contact with it, whereas the solution of the lactate of iron is composed of elements which enter into the composition of the blood; that when thrown into the living vessels it does not coagulate it, but produces a thickening of the coats and a deposition of coagulable lymph from subacute inflammation; the difference between the two results will be readily understood. It is probable that the solution of the lactate, when thrown into the vessels, immediately undergoes decomposition, the acid combining with the soda of the blood, and the base passing into a higher state of oxydation in which it naturally exists in that fluid. In no case has this solution, when thrown into the blood or into a vascular tumor, shown a tendency to produce suppuration, but a slow inflammation, of an adhesive kind and limited extent. In one case, where it was inadvertantly pressed in small quantity into the subcutaneous cellular tissue, instead of into the vein as intended, a hard swelling having all the appearance of a boil or small carbuncle was the result; but even this effect will not be produced by it, when used in that manner, unless the solution be in a certain degree concentrated.

Encouraged by the result in this case, I did not hesitate, on the first favorable opportunity, to repeat the treatment.

Case 12.—Miss P. aged 21 years, from Rockford, Ill., has had an erectile venous tumor situated on the inner side of the lower lip, near the right commissure, from a very early age. At present it is of the size of a very large nut.

Operation.—Having everted the lip and covered the tumor with a tube of suitable size, applied and exhausted as a cupping glass. I introduced a minute trocar to the center of the tumor, and injected through it a drachm of the solution of the lactate of iron, of the strength of eight grains to the ounce. After retaining the cupping glass in place for about a minute, in order to give the solution an opportunity to act upon the internal membrane of the vessels, it was removed. But slight inflammation followed—a little induration, which speedily passed over. At the end of a week no change was perceptible. This operation was repeated six times in the same manner, the quantity of solution used being greater, and the cupping being retained longer, without effecting a cure. At the end of two months the tumor was about one-half its size at the commencement, having had knobs at some points, and vessels filled with blood at others.

I considered this trial as conclusive against the lactate in this form of the disease.

6. *Ligature of the Principal Arteries Leading to the Tumor.*—Dr. Norris (*American Journal of Medical Sciences*, for July, 1847,) publishes a table in which is embraced twelve cases of ligature of the common carotid artery, for erectile tumor in the orbit. He states the result as follows: cured, 9; recovered, 2; died 1.

While admitting that ligature of the carotid artery has been more successful for tumors in the orbit than that of other arteries, for those situated elsewhere I believe that the result in those cases will be found less favorable than stated by Dr. Norris.

Mr. Wardrop had a fatal case. The tumor was seated on the side of the jaw. There are several cases on record which are said to have resulted in improvement. As these were reported for the most part within a few months after the operation, the improvement is likely to have been but temporary.

Mr. Travers tied the carotid artery for pulsating, thrilling tumor of the orbit. Two years afterwards it gave no trouble.

Boyer relates the case of a congenital pulsating tumor of the color of wine, situated over the left temporal region. Pelletier tied the temporal artery after it had been incised, without success. The patient died on the 14th day. (*Maladies Chirurgicales*, vol. ii, p. 295.)

Dupuytren is said to have tied the carotid artery in a similar case, with "partial success."

Mr. Arendts reported a case of ligature of the carotid artery for erectile tumor of the right temple, the result of a blow on a congenital nævus. The tumor burst during the operation, and eight pounds of blood were lost. The next day twelve small arteries were tied. (*Medical Chir. Transactions*, vol. 22, p. 124.

Mr. Scott, of the London Hospital tied the right carotid artery, for a tumor of the same kind, resulting, from injury. The case is said to have been improved. (*Medical Chir. Transactions*, vol. 22, p. 124.)

Prof. Mussey, in 1827, tied both carotid arteries for an erectile tumor on the vertex. These operations failing, he extirpated the tumor. (*American Journal of the Medical Sciences*, for Feb. 1830.)

Mr. Wardrop reports two other cases, one of which was successful. (*Walton's Ophthalmic Surgery*, p. 225.)

I have already reported the case of William Wells, in which the ligature of the left carotid artery failed to cure an erectile tumor of the orbit.

CASE 13—*Erectile Tissue occupying the palm of the hand—Ligature of the Radial and Ulnar Arteries—Cure.*—Mrs. E. A. G., aged 25 years, applied, February 25, 1856. A painful enlargement of the left hand. It had been gradually coming on for many years, and at this time presented the following appearances. The hand was of double its natural thickness of a florid red color, throbbed and thrilled at each arterial pulsation. All the veins and arteries were much enlarged and pulsated the arteries with much force. The hand was very tender and painful. I tied up the radial and the ulnar arteries at the wrist. They were both much enlarged and very tortuous, and their coats thin, like those of a vein.

On the second day some swelling and diffuse redness, like erysipelas, showed itself, which disappeared under appropriate treatment, and the patient left on the 7th day in the way of cure.

Oct. 1858.—This patient remained cured.

A review of the cases in which arteries have been tied for erectile tumor shows that in the accidental pulsating, thrilling sub-cutaneous variety, the operation is often successful, particularly when the disease is seated in the orbit, while in the tumors congenital variety it has little chance of success.

There are, in addition to the means already noticed, a variety of others of a miscellaneous character, which must not be entirely passed over.

Of these, vaccination is often recommended. It was first resorted to by Mr. Hodgson, of Birmingham, about the year 1828. It has been used by Messrs. Young & Auchincloss, of Glasgow. It may be used in cases of very slight mark, but even in these I have never seen it of much benefit and in more extensive or deeper seated disease it is altogether trivial and unworthy of trial.

Analagous to this plan, but more effectual, is that of applying the Tart. Ant. and Potass. It has been used by Cumming, fifteen grains mixed with one drachm of galbanum plaster, and applied upon the part affected. Sloughing is said to have followed its application. Mr. Hickman used it mixed with olive oil.

Mr. Calloway used the paste of chloride of zinc. Mr. Helmuth the arsenical paste. Mr. Lafargue, inoculation with croton oil.

I have often tried each of these, and in addition, vesication with the Spanish fly, with the nitrate of silver, creosote, and other vesicants. They all possess the property of diminishing the intensity of the redness in congenital nævus, but are not effectual except in spots of very limited size. Even in these they commonly fail.

Mr. Edwards reports two cases cured by the application of iodine paint. They were small and superficial. (*American Journal* for October, 1855.)

In a severe case, which I shall report further on, the "iodine paint" was used perseveringly and seemed to have the effect of somewhat bleaching the discolored surface, but this was temporary.

Dr. Marshall Hall recommended sub-cutaneous laceration of the diseased tissue. The recommendation is strictly in accordance with physiological views, for it is know that the blood coagulates whenever it comes in contact with a cut surface, while the internal surface of veins, arteries, or cells of erectile tissue possess the property of preserving its fluidity.

It has been carried into practice in one instance, by J. M. Warren, who employed it after using the frigoric mixture. The case was said to be discharged much improved. M. Jules Guerin used it in case of a tumor situated on the side of the nose. Two operations were performed with success. M. Blandin is said to have used it several times successfully.

Dr. Macke recommends corrosive sublimate four parts, to collodion thirty parts. He says this produces an eschar one or two lines in depth. I have tried it, but from some reason have not found it to produce any perceptible effect.

Cutting, and burning by means of a fine platinum wire, heated by a currant of galvanism is now being used. It is not easy to see what advantage can be derived from it.

Passing over a number of applications not deemed worthy of mention, I shall finish the enumeration of different methods of treating erectile tumors, by a few words on the subject of electricity or electro-puncture. M. Petroquin recommended it as early as 1845, and during the following year used it in several cases of aneurism, and Messrs. Liston and Phillips had, it is said, previously employed it without success.

M. Nelaton has reported a case of pulsating, thrilling tumor, on the bridge of the nose, the result of an injury, cured by six applications of galvanism. Two needles were applied to those points of the tumor where the pulsations were most distinct, and brought into connection with the battery of Bunsen, composed of thirty pairs of plates, which were permitted to act for ten minutes. (*Gazette Medicale* for 1846, p. 736.)

Recently it has been found by experiment, that the best method of proceeding is to use steel needles covered with zinc. These should be thrust into the tumor and connected with the positive pole, after which the negative pole should be connected with a piece of platinum and placed on the skin near the aneurism, the integument having been previously moistened with a saline solution. (*American Journal* for April, 1854, p. 507.)

Neither experience no reasoning on the subject have as yet given a very favorable impression of this treatment in erectile tumors. It must, however, be admitted that as yet it has not been properly tested, and that it is well deserving a further trial.

The surgeon, after taking a survey of all the means which have been enumerated, and considering their relative value and efficiency, will be induced to confine his choice within a very limited range, and to combine them judiciously, rather than attach himself obstinately to a single plan. I may, perhaps, best illustrate my own practice by a report of cases treated. The following is one of the most formidable which has fallen under my care, and is, in my judgment, one of those in which palliation is all that is to be aimed at.

CASE 14.—B. E., aged twenty years, has an erectile tumor occupying the region of the neck and face. The left side of the tongue, the gums of the upper and lower jaws, the whole thickness of the lip and cheek, were very vascular, pulsating but not thrilling. The disease was congenital, but had increased with great rapidity after an injury which he had received some two years previously.

In Jan., 1857, the treatment was commenced by inserting fine steel needles vertically through the lower lip, from its edge to the chin, one at each angle of the mouth, and the others at regular distances between. The needles were placed very near the mucous membrane, and ligatures tied firmly upon them, in form of a figure eight, and then irregularly from one to the other. At the end of five days the tissues thus embraced had been very nearly cut through by the ligatures. They and the needles were therefore removed, cicatrization proceeded, and at the end of three weeks the lip had been greatly reduced in size, and was traversed in different directions by fine bands of cicatrix. As there were still points at which the disease existed in the lower lip, these were punctured at different times by the cauterizing needles, at a white heat, the patient being under the influence of chloroform. The lip having been thus greatly reduced in size, the gums were next attacked.

The patient having been placed under the influence of chloroform, cauterization with the iron at a white heat, was effected on the whole of the diseased gums, in a very thorough manner. While the cicatrization of these was proceeding, I made use of means for diminishing the intense bluish-red color of the surface.

Vesication with blistering collodion, nitrate of silver, creosote, strong acids, and various other irritants were resorted to. Strong solutions of iodine were also used. The plan pursued was to commence at the edge and even let the vesication extend a little upon the sound skin and about an inch in breadth and three inches in length upon the diseased surface. This was kept up for about two weeks, when, as it was healing, another strip was subjected to a similar process. The patient submitted himself to this treatment with admirable fortitude for six months, at the end of which time the swelling was greatly diminished, particularly that of the lip, which applied itself to the teeth, very perfectly, and the color of the face was in many parts scarcely greater than that in many persons of very florid complexion.

I presume that many surgeons would, in this case, have resorted to ligature of the carotid artery, but aside from its liability to fail of success in any similar case it is an operation of considerable danger and the necessity for it was not sufficiently urgent in my view to justify its performance. The treatment resorted to, although long, was not dangerous, and resulted in an amelioration extremely gratifying and satisfactory.

The relative merits of the different methods of treating erectile tumors, may be summed up as follows:

I. Excision should be performed in every case where the size and situation of the tumor will admit of its being performed. This is almost as much a rule in these cases as in cancer. The exceptions are the slight cases which may be trusted without treatment until they increase in size.

II. When excision would cause too great a loss of substance, danger from hemorrhage, or when, from any cause, excision is objected to, strangulation is to be preferred next in order, and whether effected with ligature alone, or with needles, or other means, it should always, if possible, embrace the whole diseased structure.

III. In limited superficial naevi and erectile tumors, particularly if placed over bony surfaces, compression will often diminish, if not cure, the disease.

IV. In deep-seated tumors, particularly aneurisms by anastomosis, cauterization with the hot needles is an extremely efficient remedy, either by itself or in connection with other means.

V. Setons or metallic needles may be used in the venous forms of the disease. They are more effectual when placed, to some extent, in sound tissue.

VI. Ligature of the principal artery leading to the part, is adapted to the variety called aneurism by anastomosis, the accidental thrilling variety, and particularly to that variety situated in the orbit of the eye. I believe, however, that it is more dangerous and less necessary than is generally supposed.

VII. Vesicants, escharotics and caustics, are adapted to complete a cure, when a small portion of tissue remains after excision, strangulation or seton. They are uncertain and little to be relied on.

VIII. A combination of several of these methods of treatment will often be found advisable.

Note.—Since writing the above, I have received an interesting paper, published in the *Montreal Chronicle*, by John Ridleh, M. D. Dr. Ridleh injected four erectile tumors of different kinds with perchloride of iron, and produced sloughing in all. This, although effectual in each instance, is to be regarded as only a different way of using caustic, the advantages of which require, perhaps, further observations before pronouncing upon.

